# Genetic association between NFKB1 −94 ins/del ATTG Promoter Polymorphism and cancer risk: a meta-analysis of 42 case-control studies

**DOI:** 10.1038/srep30220

**Published:** 2016-07-22

**Authors:** Duan Wang, Tianhang Xie, Jin Xu, Haoyang Wang, Weinan Zeng, Shuquan Rao, Kai Zhou, Fuxing Pei, Zongke Zhou

**Affiliations:** 1 West China Hospital/West China School of Medicine, Sichuan University, Chengdu, 610041, China; 2 Tianjin Hospital, Tianjin, 300211, China; 3 Department of Orthopaedics, West China Hospital/West China School of Medicine, Sichuan University, Chengdu, 610041, China; 4 Center for Joint Surgery, Southwest Hospital, Third Military Medical University, Chongqing, 400038, China; 5 School of Life Science and Engineering, Southwest Jiaotong University, Chengdu, 610031, China

## Abstract

Accumulating evidences have indicated that the functional *-94 ins/del ATTG* polymorphism in the promoter region of human nuclear factor-kappa B1 (*NFKB1*) gene may be associated with cancer risk. However, some studies yielded conflicting results. To clarify precise association, we performed a comprehensive meta-analysis of 42 case-control studies involving 43,000 subjects (18,222 cases and 24,778 controls). The overall results suggested that the *-94 ins/del ATTG* polymorphism had a decreased risk for cancer, reaching significant levels in five genetic models (dominant model: OR = 0.86, 95% CI = 0.79–0.95, P = 0.002; recessive model: OR = 0.84, 95% CI = 0.74–0.94, P = 0.003; homozygous model: OR = 0.77, 95% CI = 0.66–0.90, P = 0.001; heterozygous model: OR = 0.90, 95% CI = 0.83–0.98, P = 0.011; allelic model: OR = 0.89, 95% CI = 0.83–0.96, P = 0.002). Furthermore, the *-94 ins/del ATTG* polymorphism could confer a decreased or increased risk for cancer development among Asians and Caucasians, respectively. Additionally, the stratification analysis revealed a significant association between the variant and decreased risk of oral, ovarian, and nasopharyngeal cancer in Asians. After we adjusted p values using the Benjamini-Hochberg false discovery rate method to account for multiple comparisons, these associations remained.

Characterized by angiogenesis and inflammation, cancer is a major public health problem and a complex terminal disease worldwide, which is injurious to human health and lives^1^,^2^. With the aging population together with increasing formation of cancer-causing habits, it is estimated that there would be more than 22 million new cases suffering from cancer in the next decades. Therefore, the clinical and economic burden of cancer on healthcare will be heavier^2^,^3^. Although the mechanism of carcinogenesis has not been completely clarified, abundant evidences indicated that a combination of genetic predisposition and environmental exposures is believed to contribute to pathogenesis of cancer^4^. With numerous recent advances in genetic research, many genes that are related to malignancy susceptibility have been identified as candidates in multiple populations, especially nuclear factor-kappa B 1 (*NFKB1*)^5^,^6^,^7^,^8^,^9^,^10^.

There is mounting evidence that inflammatory cytokines and carcinogens closely related to cancer development are involved in activating the common cell survival signaling pathways^11^,^12^. NF-κB serves as a major cell survival signal and is involved in multiple steps in carcinogenesis and in cancer cell’s resistance to chemo- and radiotherapy^12^,^13^. It was identified as an inducible transcriptional factor binding to the intronic enhancer of the kappa light chain gene in every cell type^14^,^15^. Subsequently, NF-κB acts as an important regulator of more than 200 genes known to be involved in cell adhesion, cell survival, inflammation, apoptosis, and growth^16^,^17^. The NF-κB family is composed of five members in mammal: p50/p105, p65/RelA, c-Rel, RelB, and p52/p100 (NFKB2), which can form different dimeric combinations. *NFKB1* gene, mapped to chromosome 4q24, encodes p105/p50 isoforms of NF-κB, involved in cancer initiation and progression. The *-94 insertion/deletion* (ins/del) *ATTG* polymorphism within the promoter region of the *NFKB1* gene could potentially affect the transcription of the gene and the function of NF-κB protein, sequentially leading to loss of binding capacity to nuclear proteins and reduced promoter activity^18^.

The *-94 ins/del ATTG* (rs28362491) polymorphism that involves deletion of four nucleotides in the promoter region of *NFKB1* gene consists of three genotypes: homozygote insertion or wild-type (WW), homozygote deletion or variant (DD), and heterozygous ins/del (DW)^7^,^18^. According to published data, several search groups have investigated the relationship between the polymorphism and cancers risk^7^,^10^,^19^,^20^,^21^,^22^,^23^,^24^,^25^,^26^,^27^. However, these results obtained remains inconsistent. For instance, numerous studies reported that the del/del genotype of the polymorphism could contribute to an increased colorectal cancer (CRC)^28^,^29^,^30^, papillary thyroid carcinoma (PTC)^24^ and lung cancer (LC)^13^ in Chinese or Caucasian populations, while some other studies showed that the ins/ins genotype of the polymorphism could increase the risk of CRC^20^,^31^, LC^26^ and bladder cancer (BC)^32^ in different populations. In addition, several studies showed a lack of relationship between the polymorphism and CRC risk^7^.

Up to now, some earlier meta-analyses based on different strategies have tried to investigate the link between the polymorphism and cancer risk. Yang *et al*.^33^ and Nian *et al*.^34^ reported that the *-94 ins/del ATTG* polymorphism was significantly associated with decreased cancer risk in Asian populations, but not in Caucasian populations. Unfortunately, the two previous meta-analyses included some studies, in which the genotype distributions of control groups were not consistent with Hardy–Weinberg equilibrium (HWE). In addition, obviously high heterogeneity was detected under all five genetic models in their studies, but they only conducted subgroup analysis based on ethnicity to investigate the origin of the high heterogeneity. Furthermore, the authors did not perform tests to access statistical significance of multiple null hypotheses^35^. Henceforth, some new studies further assessed the relationship between the polymorphism and cancer risk among multiple ethnic populations. However, the association remains inconclusive due to the inconsistent results from the published publications^23^,^24^,^25^,^26^,^27^,^30^,^36^,^37^,^38^,^39^,^40^,^41^,^42^. Therefore, the data need to be updated and more reliable evaluations of the polymorphism with cancer risk are warranted.

Due to the inconsistency of past studies and the critical role of the variant in the pathogenesis of cancer, we conducted an updated meta-analysis to investigate the association of *NFKB1 -94 ins/del ATTG* promoter polymorphism and cancer risk by precise results.

## Materials and Methods

### Study selection

A systematic search of PubMed, Embase, ISI Web of Science, and Chinese National Knowledge Infrastructure (CNKI) database was conducted by two independent reviewers to identify relevant articles, which had investigated the association between *NFKB1 -94 ins/del ATTG* polymorphism and susceptibility to cancer (latest electronic search was updated on March 10, 2016). The search terms were used as follows: ‘cancer’ or ‘carcinoma’ or ‘tumor’ or ‘carcinogenesis’ or ‘neoplasm’ And ‘NF-κB’ or ‘Nuclear factor-κB 1’ or ‘NFKB1” And ‘polymorphism’ or ‘variant’ or ‘mutation’ or ‘polymorphisms’ or ‘variants’ or ‘mutations’. No publication date or languages restrictions were imposed. Studies eligible for this meta-analysis must fulfill the following inclusion criteria: (a) studies using a case-control design; (b) studies evaluating the association between *NFKB1 -94 ins/del ATTG* polymorphism and cancer risk; (c) genotype distributions should be available for estimating the odds ratios (OR) with 95% confidence interval (CI); (d) the genotype distributions among control groups must be consistent with HWE. For multiple reports based on overlapping samples, only the most recent study or the study with the largest number of subjects were retained. Exclusion criteria were defined as follows: (a) abstracts, conferences, reviews, letters, case reports, and non-human studies; (b) repeated or overlapping publications; (c) studies reporting neither genotype distributions nor allele frequency; (d) the studies that do not follow HWE. We also inspected the references list of reviews or previous meta-analyses for potentially relevant publications. Two investigators independently screened all abstracts and citations to extract potentially eligible studies.

### Data extraction

Two investigators independently collected the information of each eligible study based on the inclusion and exclusion criteria. First author, publication year, country, ethnicity, control source, genotyping technology, genotype and allele distributions, total number of cases and controls, and cancer type were extracted. In case of discrepancies, we checked the collected data and reached a consensus through discussion to ensure accuracy of the data. The information is shown in Tables 1 and 2.

### Quality score assessment

The quality of included studies was estimated according to the Newcastle–Ottawa Scale (http://www.ohri.ca/programs/clinical_epidemiology/oxford.asp). The scale use a ‘-’ rating system to evaluate quality based on three points including the selection of the study groups, comparability of the groups, and the ascertainment of the exposure or outcome of interest in the case-control study. Total scores range from 0 to 9 points. A study with scores of more than 7 points was considered to be a high quality study (Table 1). Discrepancies would be resolved by discussion with a third author until a consensus was reached.

### Statistical method

In this meta-analysis, all of the data in eligible studies were calculated as odds ratios (OR) with 95% confidence interval (CI) to assess the strength of association between *NFKB1 -94 ins/del ATTG* promoter polymorphism and the risk of cancer. The *-94 ins/del ATTG* polymorphism consists of three genotypes: homozygous insertion or wild-type (WW), homozygous deletion or variant (DD), and heterozygous ins/del (DW). The pooled OR was estimated in five genetic models: (1) dominant model: del/del + del/ins versus ins/ins (DD+DW vs. WW); (2) recessive model: del/del versus del/ins + ins/ins (DD vs. DW+WW); (3) homozygous model: del/del versus ins/ins (DD vs. WW); (4) heterozygous del/ins versus ins/ins (DW vs. WW); and (5) allelic model: deletion allele versus insertion allele (D vs. W), which was considered statistically significant when the P value of the Z test was less than 0.05.

Heterogeneity among studies was evaluated by Chi-squared-based Q-test and I^2^ statistics, which confirmed the heterogeneity at *P*-value < 0.10. If no or low heterogeneity existed (*P* < 0.10), the random-effects model (REM) was applied to estimate pooled OR. Otherwise, the fixed-effects model (FEM) was used. Moreover, HWE was recalculated in control groups by Pearson’s *χ*^*2*^ test before this meta-analysis was conducted. To explore the potential heterogeneity among studies, we conducted subgroup analysis based on ethnicity (Asian and Caucasian), control source (hospital-based (HB) and population-based (PB) population), genotyping method (TaqMan and others), quality score of studies, and type of cancer. In addition, sensitivity analyses were also conducted through sequentially excluded individual studies to investigate the potential origin of heterogeneity and to assess the stability of the results.

Potential publication bias was tested by several methods. Visual inspection of asymmetry in funnel plots was carried out. Furthermore, Egger’ regression and Begg’s test were also utilized to detect publication bias, and *P* < 0.05 was considered statistically significant. Benjamini–Hochberg false discovery rate (FDR) test was used to correct for multiple comparisons yielding P_corr_ value less than 0.05 was considered as significant. All statistical analyses were performed with STATA 12.0 software (Stata Corp LP, College Station, TX).

## Results

### Study inclusion and characteristics

**Figure 1:**
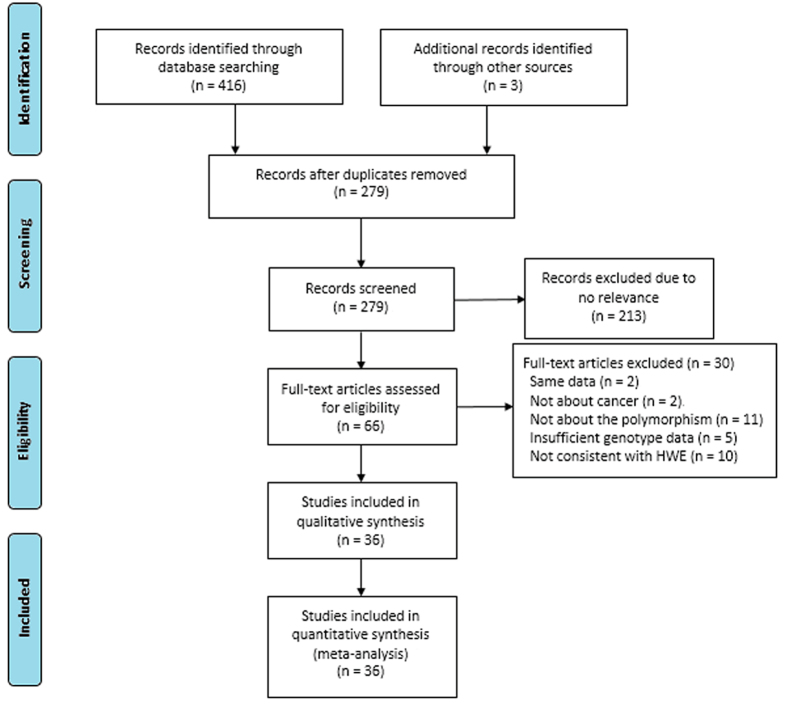
Flow diagram of literature selection and data extraction for meta-analysis.

This meta-analysis design was based on the Preferred Reporting Items for Systematic Reviews and Meta-Analyses (PRISMA) prospectively. The process of study selection was showed in Fig. 1. We totally identified 419 articles with our search strategy. After removing duplications, scanning titles and abstracts and reading the full-text, 36 articles were eligible based on our inclusion and exclusion criteria in this meta-analysis. Among these selected articles, Riemann *et al*.^43^ and Li *et al*.^39^ investigated the link between the *-94 ins/del ATTG* polymorphism and the risk of three different cancer types, respectively. Therefore, we treated them as six separate comparisons. In addition, Huang *et al*.^44^ and Liu *et al*.^41^ conducted their research in two different places (eastern and southern china), which were treated as four individual comparisons. Therefore, a total of 42 studies from 36 articles were identified, including 43,000 subjects (18,222 cases and 24,778 controls)^8^,^10^,^13^,^19^,^20^,^21^,^22^,^23^,^24^,^25^,^26^,^27^,^28^,^29^,^30^,^32^,^36^,^37^,^38^,^39^,^40^,^41^,^42^,^43^,^44^,^45^,^46^,^47^,^48^,^49^,^50^,^51^,^52^,^53^,^54^,^55^. Of the 42 included studies, five studies involving 7,186 subjects reported on colorectal cancer (CRC). In the remaining studies, there were two studies on renal cell carcinoma (RCC), four studies on bladder cancer (BC), two studies on gastric cancer (GC), three studies on hepatocellular carcinoma (HCC), four studies on prostate cancer (PC), four studies on ovarian cancer (OC), five studies on lung cancer (LC), three studies on nasopharyngeal carcinoma (NPC), two studies on oral squamous cell carcinoma (OSCC). Other cancers, such as multiple myeloma, breast cancer, cervical squamous cell carcinoma, acute myeloid leukaemia, were relatively less investigated and thereby merged into “other cancer” category. In addition, there were 30 studies for the Asian population and 12 studies for the Caucasian population. As for control source, 10 studies employed PB control, while 29 studies conducted their studies applying HB control. Moreover, the estimated quality of each study was in the range of 6–9 points. The genotype distributions for control populations in all studies were conformed to HWE. Characteristics of included studies are shown in Tables 1 and 2.

### Quantitative data synthesis

**Figure 2:**
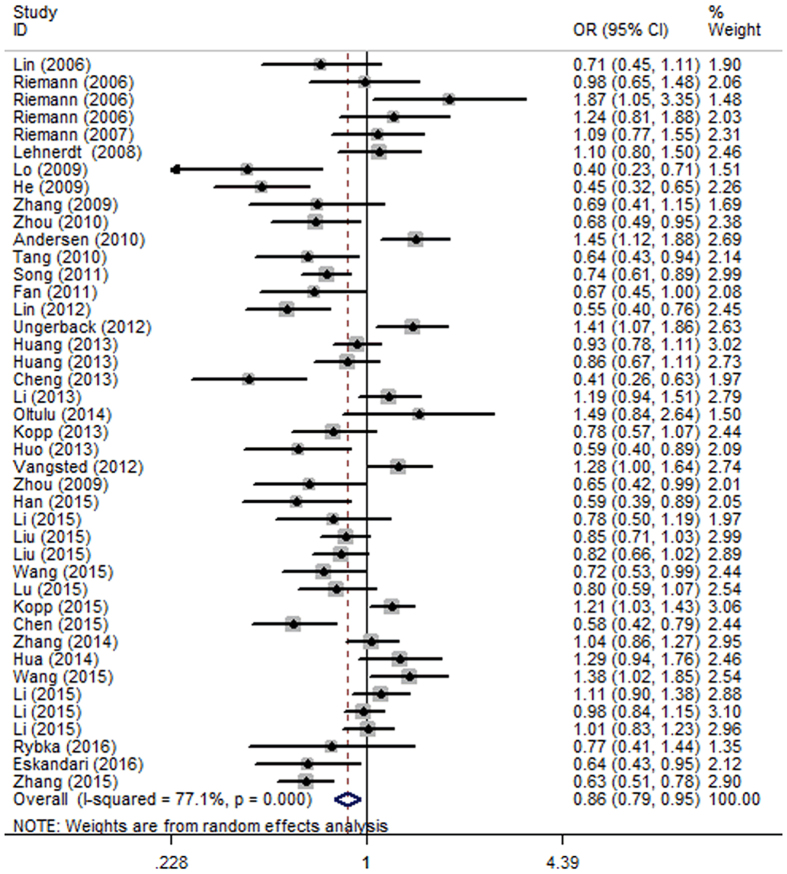
Forest plot of cancer risk associated with NFKB1 -94 ins/del ATTG polymorphism in dominant model (DD+DW vs. WW) for the overall population.

The meta-analysis results have been summarized in Table 3. For the presence of moderate heterogeneity across all levels of analysis, random-effects model (REM) was applied to calculate the summary odds ratios (OR) in all five genetic models (Table 4). Overall, a significantly decreased risk of cancer was associated with *NFKB1 -94 ins/del ATTG* polymorphism in all five genetic models (DD+DW vs. WW: OR = 0.86, 95% CI = 0.79–0.95, *P* = 0.002; DD vs. DW+WW: OR = 0.84, 95% CI = 0.74–0.94, *P* = 0.003; DD vs. WW: OR = 0.77, 95% CI = 0.66–0.90, *P* = 0.001; DW vs. WW: OR = 0.90, 95% CI = 0.83–0.98, *P* = 0.011; D vs. W: OR = 0.89, 95% CI = 0.83–0.96, *P* = 0.002) (Fig. 2). In addition, corrected *P* values for multiple testing remained significant (Table 4).

### Subgroup analysis

To investigate the moderate heterogeneity in the outcomes and the inference of the studies, we pooled the odds ratios (OR) and 95% confidence interval (CI) from further subgroup analyses of ethnicity, control source, genotyping method, cancer type, and quality score. When stratified by control source, REM was utilized due to the presence of heterogeneity in all above genetic models (Table 4). Consistently, *NFKB1 -94 ins/del ATTG* polymorphism was significantly associated with decreased cancer susceptibility among HB population in all genetic models, among which the most obvious is for dominant model (DD+DW vs. WW: OR = 0.85, 95% CI = 0.77–0.95, *P* = 0.003), and weakest for recessive model (DD vs. DW+WW: OR = 0.86, 95% CI = 0.74–0.99, *P* = 0.04). Corrected *P* values for multiple testing remained significant. However, no such significant relationship was detected for PB population in any genetic model (Table 3).

**Figure 3:**
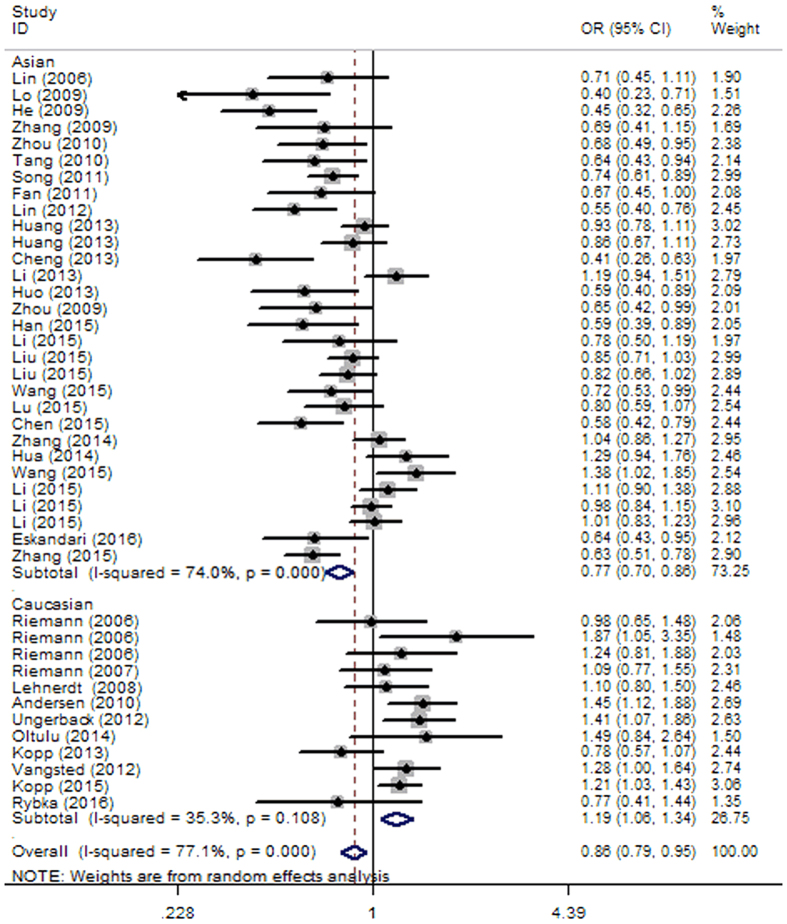
Forest plot of cancer risk associated with NFKB1 -94 ins/del ATTG polymorphism in dominant model (DD+DW vs. WW) for the subgroup analysis by ethnicity (Caucasian and Asian).

Subgroup analysis based on ethnicity was also conducted in this analysis. Since moderate heterogeneity was observed in all genetic models, we used the REM of analysis. The overall results showed that a significantly strong association between *NFKB1 -94 ins/del ATTG* polymorphism and decreased cancer risk was seen for Asian population in all genetic models (DD+DW vs. WW: OR = 0.77, 95% CI = 0.70–0.86, *P* < 0.0001; DD vs. DW+WW: OR = 0.77, 95% CI = 0.67–0.90, *P* = 0.001; DD vs. WW: OR = 0.66, 95% CI = 0.55–0.80, *P* < 0.0001; DW vs. WW: OR = 0.85, 95% CI = 0.81–0.90, *P* < 0.0001; D vs. W: OR = 0.82, 95% CI = 0.76–0.90, *P* < 0.0001) (Fig. 3). In contrast, the Caucasian population exhibit *NFKB1 -94 ins/del ATTG* promoter variant associated increased susceptibility to cancer in dominant (DD+DW vs. WW: OR = 1.20, 95% CI = 1.10–1.31, *P* < 0.0001), homozygous (DD vs. WW: OR = 1.15, 95% CI = 1.01–1.31, *P* = 0.03), heterozygous (DW vs. WW: OR = 1.22, 95% CI = 1.11–1.34, *P* < 0.0001), and allelic (D vs. W: OR = 1.11, 95% CI = 1.04–1.17, *P* = 0.001) models (Table 3), with low heterogeneity found in Caucasians (Table 4). Moreover, no significant variables were identified after corrections for multiple comparisons.

Subgroup analysis was conducted among 6 methods for genotyping. REM was used since moderate heterogeneity was identified in this subgroup analysis. In the polymerase chain reaction-restriction fragment length polymorphism (PCR-RFLP) group and polymerase chain reaction-polyacrylamide gel electrophoresis (PCR-PAGE) group, significantly decreased association between *NFKB1 -94 ins/del ATTG* promoter variant and cancer risk were identified, among which the most obvious is for recessive model in the two groups (PCR-RFLP group: DD vs. DW+WW: OR = 0.72, 95% CI = 0.60–0.87, *P* = 0.001; PCR-PAGE group: DD vs. DW+WW: OR = 0.65, 95% CI = 0.50–0.85, *P* = 0.002), respectively. In contrast, no relationship was detected in TaqMan, MassARRAY, PCR-CGE, and Pyrosequencing group (Table 3). When stratified by quality score, significant decreased association was only identified in the group whose quality score ≤ 7 points in dominant (DD+DW vs. WW: OR = 0.86, 95% CI = 0.76–0.97, *P* = 0.013), recessive (DD vs. DW+WW: OR = 0.81, 95% CI = 0.71–0.91, *P* = 0.001), homozygous (DD vs. WW: OR = 0.75, 95% CI = 0.63–0.89, *P* = 0.001), and allelic (D vs. W: OR = 0.87, 95% CI = 0.80–0.95, P = 0.002) model (Table 3).

In addition, stratification analysis by cancer type was also performed. Further analysis showed that the substitution was significantly related to decreased risk of OSCC (DD+DW vs. WW: OR = 0.60, *P* < 0.0001; DD vs. DW+WW: OR = 0.63, *P* < 0.0001; DD vs. WW: OR = 0.49, *P* < 0.0001; DW vs. WW: OR = 0.67, *P* = 0.004; D vs. W: OR = 0.70, *P* < 0.0001), OC (DD+DW vs. WW: OR = 0.67, *P* < 0.0001; DD vs. DW+WW: OR = 0.68, *P* = 0.005; DD vs. WW: OR = 0.54, *P* < 0.0001; DW vs. WW: OR = 0.73, *P* = 0.001; D vs. W: OR = 0.75, *P* < 0.0001), and NPC (DD+DW vs. WW: OR = 0.82, *P* = 0.003; DD vs. DW+WW: OR = 0.74, *P* < 0.0001; DD vs. WW: OR = 0.63, *P* < 0.0001; D vs. W: OR = 0.83, *P* < 0.0001) in genetic models. Moreover, significantly decreased relationship between the polymorphism and LC risk was only found in heterozygous model (DW vs. WW: OR = 0.84, 95% CI = 0.74–0.96, *P* = 0.008). In addition, corrected p values for multiple testing remained significant (Table 4). Nevertheless, there was no significant relationship between the polymorphism and the remaining cancer types in any genetic model, including CRC, RCC, BC, GC, HCC, PC and other cancer (Table 3).

### Publication bias

**Figure 4:**
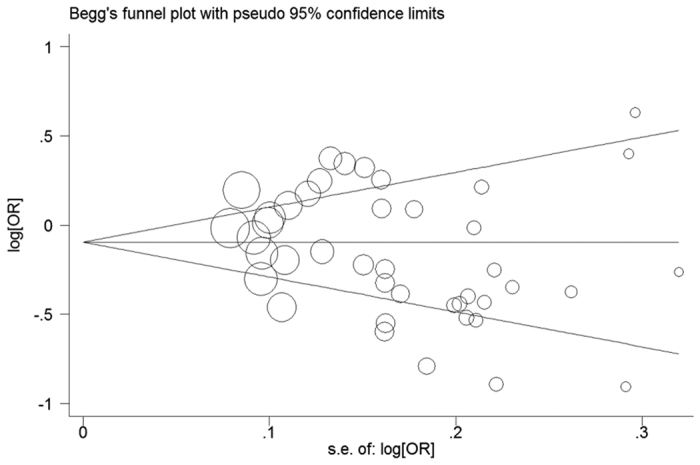
Begg’s funnel plot of publication bias test for the association between NFKB1 -94 ins/del ATTG polymorphism and cancer risk under dominant model (DD+DW vs. WW).

Publication bias was assessed through the Begg’s funnel plot and Egger’s regression intercept tests. The shape of the Begg’s funnel plot did not reveal basically asymmetric distribution in all comparisons of the overall population. Moreover, the result of Egger’s test (dominant model: *P* = 0.269; recessive model: *P* = 0.202; homozygous model: *P* = 0.133; heterozygous model: *P* = 0.471; allelic model: *P* = 0.245) further provided no evidence of significant publication bias. Our observation of symmetric funnel plots and non-significant statistical tests confirmed no publication bias (Fig. 4).

### Sensitivity analyses

To validate our results, we further conducted sensitivity analyses to evaluate the stability of these results in this meta-analysis. Firstly, the random-effects model was compared with the fixed-effects model, and the statistically similar results were obtained in all genetic models. Secondly, we applied leave-one-out method by sequentially excluding each study to assess the influence of individual study on the obtained conclusions. The conclusions remained unchanged in all comparisons, suggesting the stability of our meta-analysis. Furthermore, due to the higher number of studies of Asian population, we conducted the analysis with randomly selecting 50% of the case-control studies for many times to determine the sensitivity. However, the corresponding pooled ORs were generally similar in each comparison (data not shown). Therefore, our conclusion in this meta-analysis was stable and credible.

## Discussion

The NF-κB family constitutes a class of pleiotropic transcription factors, which seems to contribute to tumor angiogenesis and progression by regulating the transcription of genes involved in immune response, cell proliferation, apoptosis, and angiogenesis^17^,^56^. The NF-κB family members can form homo-dimmers and hetero-dimmers to produce more than 15 distinct active complexes, among which p50/p65 heterodimer is one of the most sufficient forms and is present in almost all cell types^57^. NF-κB complexes are constitutively active in various cancer cell lines and tissues, indicating the involvement of NF-κB in tumorigenesis^58^,^59^. In addition, various inflammatory cytokines, such as TNF-a, IL-6, and IL-8, regulated their biological effects by activating NF-κB. In consequence, the abnormality and disturbance of NF-κB function can reduce diverse biological processes, including uncontrolled cell proliferation and suppression of apoptosis, thereby rendering a cell more prone to malignant transformation^31^.

A functional *-94 ins/del ATTG* (*rs28362491*) polymorphism in the promoter region of the p50/p105-encoding *NFKB1* gene located between two received key promoter regulatory elements modulates its transcription and then affects the production of protein subunits^18^,^31^. Previous studies have shown that the -94del allele may result in significantly decreased *NFKB1* transcript level and hence, reduce p50/p105 protein production^18^,^20^. Therefore, the different level of the protein subunits between the carriers of -94del allele and -94ins allele may be expected to contribute to inter-individual variations in cancer risk.

In this meta-analysis, we investigated the *-94 ins/del ATTG* on *NFKB1* gene locus with 42 separate case-control studies (18,222 cases and 24,778 controls) focusing on the relationship of this variant to cancer risk. The overall results revealed a significant association between -94 del/del variant genotype carriers and decreased cancer risk. Despite the mechanism remains unclear, considering the critical role of NF-κB in various biological pathways, we could hypothesize that ATTG deletion causes the loss of binding to nuclear proteins, which leads to reduced promoter activity^13^. The promoter sequence with -94 del allele results in lower transcriptional activity and thereby causes decreased levels of p50/p105 in the cytoplasm, which serves a critical function in trans-activating anti-apoptosis genes and inhibiting cell apoptosis, thereby promoting cellular proliferation. In addition, the p50 in -94 del carriers can form lesser heterodimers with p65 to mediate the inflammatory pathway compared to the -94 ins allele carriers. Therefore, the -94 del allele may play a protective role in cancer risk.

However, we must treat these results cautiously when referring to the findings, because moderate heterogeneity was detected in this meta-analysis, which may be attributed to different characteristics of the cohort, environmental exposure, clinical information and intrinsic complexity of cancer architecture. In addition, there are 30 studies for the Asian population and 12 studies for the Caucasian population. The high proportion of over-represented studies of the Asian population in the combined analysis clearly resulted in a bias, which affected the overall results. Therefore, we conducted a subgroup analysis to identify the origin of heterogeneity by ethnicity, control source, genotyping method, cancer type, and quality score.

When analysis was restricted to ethnicity, the variant del allele could confer a decreased or increased risk of developing cancer to its carriers among Asians and Caucasians, respectively. Natural selection and balance to other genetic variants may result in different genotype frequencies in different ethnicities. In addition, accumulated evidence demonstrated that individuals with the same genotype living in the different areas with different environments and life style might have a different cancer risk^7^,^20^,^31^. Therefore, this discrepancy between Caucasians and Asians could be attributed to the possible differences in genetic background, gene-environment, and non-genetic risk factors, such as smoking and high intake of salted foods. In populations where the -94del allele was identified to increase cancer risk, a plausible interpretation for such observation could be that the p50 protein lacks the C-terminal transactivation domain and may form inhibitory homo-dimers, which can serve as a role in repressing pro-inflammatory genes and then resulting in decreased levels of p50/p50 repressive homo-dimers in -94 del allele carriers^60^,^61^. Consequently, -94 del allele carriers may be genetically with a higher inflammatory response. Interestingly, the results were contrary to the results of two previous meta-analyses^33^,^34^ that no significant association was seen between *NFKB1 -94 ins/del ATTG* polymorphism and the risk of cancer in Caucasians. The contradictory results are interpreted by the following reasons: (1) the small sample was included in the previous two meta-analyses. Therefore, the results may reduce the power to reveal a reliable relationship. There were twelve case-control studies eligible for this meta-analysis among Caucasians, so the result may be closer to the real value; (2) the previous studies included many studies, which do not follow HWE. These studies may make the result incredible; (3) the sensitivity analysis was conducted through three different methods in this meta-analysis, and the results were consistent with the previous results, suggesting the results of this study were stable; (4) different clinicopathological features of cases included, different experimental designs and genotyping methods utilized in these studies; (5) we used Benjamini–Hochberg procedures to adjust for multiple comparisons in setting the level of significance. There were no significant variables after corrections for multiple comparisons.

The stronger and significantly decreased risk of cancer when analysis was restricted to hospital-based (HB) group was observed in all genetic model, but such association was not seen in population-based (PB) group. Interestingly, it was noted that moderate heterogeneity appeared in the HB group, which may result from selection bias. Additionally, HB controls were selected from hospital, which may not validly represent the exposure situation of the overall population. Therefore, results from the PB controls were thought to be more reliable.

Cancer was an overly broad collection of different types, which may have confounding factors to not reveal a reliable association. Therefore, further stratification analysis by cancer type was also conducted. The decreased risk of cancer also remained in the subgroups of oral, ovarian, and nasopharyngeal carcinoma in Asian populations. Moreover, the clinical and demographic information are essential factors affecting the overall results. Zhang *et al*.^26^ and Wang *et al*.^25^ reported that the increased risk of *-94 ins/del ATTG* polymorphism remained when analyses were done according to gender and smoking status in lung cancer among Asian population. The study by Oltulu *et al*.^13^ demonstrated that gender and age associated with lung cancer among Caucasian population. Additionally, Han *et al*.^38^ demonstrated that the impact of the -*94 ins/del ATTG* polymorphism was not present in nonsmokers and younger subjects in prostate cancer. Consequently, only increasing the number of populations, without taking into clinical and demographic information into account, will not guarantee the validity of the results. We tried to collect some subsets of clinical information and demographic data from the eligible publications to conduct further analysis. However, on one side, the relatively small sample size could reduce the statistical power of this study and result in unreliable association. On the other side, these data were collected from various cancers and ethnicities, which may give rise to confounding variables to reveal an unreliable association. Therefore, in my opinion, a better result can be given exclusively from a case-control study of large sample size, the same cancer type, and the same ethnicity.

We note several potential limitations of this meta-analysis when interpreting these results. First, we were unable to perform further analysis to investigate other risk factors of cancer risk for insufficient original data, such as age, tumor location, and gene-environment/gene-gene interaction. Second, moderate between-study heterogeneity was checked in some genetic comparisons. Further subgroup analysis showed that cancer type and control source might be the source of heterogeneity. Nevertheless, there is other inexplicable heterogeneity affecting the results. Third, publication bias might have occurred for only including published articles. Some relevant published studies or unpublished studies with null results were missed, which may affect our results. Fourth, many studies included in this meta-analysis were performed in Asian and Caucasian populations, so the association needs to be verified in other ethnicities. Despite these limitations, we created a strict protocol, and performed study selection, data identification and statistical analysis to reduce potential bias through the whole process. Thus, the objectivity and reliability of the results are guaranteed.

In conclusion, to the best of our knowledge, this is a comprehensive study to assess the relationship between *NFKB1 -94 ins/del ATTG* polymorphism and cancer risk. Our results indicated that the *-94 ins/del ATTG* polymorphism was significantly associated with decreased cancer risk in Asian populations, but increased cancer risk in Caucasian populations. However, due to the limitations of this current meta-analysis, the conclusion might be not conclusive. Therefore, in the future, there will be a need for larger sample size and more ethnic groups to further confirm the results of this meta-analysis.

## Additional Information

**How to cite this article**: Wang, D. *et al*. Genetic association between NFKB1 −94 ins/del ATTG Promoter Polymorphism and cancer risk: a meta-analysis of 42 case-control studies. *Sci. Rep.* **6**, 30220; doi: 10.1038/srep30220 (2016).

## Figures and Tables

**Table 1:** Characteristics of 42 included studies in this meta-analysis.

Author	Year	Country	Ethnicity	Cases/Control	Sample size	Control source	Cancer type	Genotyping method	Quality score
Lin	2006	China	Asian	212/201	413	HB	OSCC	PCR-PAGE	6
Riemann	2006	Germany	Caucasian	139/307	446	HB	CRC	pyrosequencing	7
Riemann	2006	Germany	Caucasian	72/307	379	HB	BCCLL	pyrosequencing	7
Riemann	2006	Germany	Caucasian	140/307	447	HB	RCC	pyrosequencing	7
Riemann	2007	Germany	Caucasian	242/307	549	HB	BC	pyrosequencing	7
Lehnerdt	2008	Germany	Caucasian	364/307	671	HB	HNSCC	PCR-RFLP	7
Lo	2009	China	Asian	182/116	298	HB	GC	PCR-RFLP	6
He	2009	China	Asian	202/404	606	HB	HCC	PCR-RFLP	7
Zhang	2009	China	Asian	117/143	260	HB	PC	PCR-PAGE	8
Zhou	2010	China	Asian	233/365	598	Mixed	CSCC	PCR-PAGE	7
Andersen	2010	Denmark	Caucasian	378/756	1134	PB	CRC	TaqMan	8
Tang	2010	China	Asian	207/228	435	HB	BC	PCR-PAGE	7
Song	2011	China	Asian	1,001/1,005	2006	PB	CRC	PCR-RFLP	8
Fan	2011	China	Asian	179/223	402	PB	OC	PCR-CGE	7
Lin	2012	China	Asian	462/520	982	HB	OSCC	TaqMan	8
Ungerback	2012	Sweden	Caucasian	348/622	966	PB	CRC	TaqMan	8
Huang	2013	China	Asian	1056/1056	2112	HB	LC	TaqMan	7
Huang	2013	China	Asian	503/623	1126	HB	LC	TaqMan	7
Cheng	2013	China	Asian	135/520	655	HB	HCC	TaqMan	8
Li	2013	China	Asian	609/640	1249	HB	BC	TaqMan	8
Oltulu	2014	Turkey	Caucasian	95/99	194	NA	LC	PCR-RFLP	7
Kopp	2013	Denmark	Caucasian	334/334	668	PB	PC	TaqMan	8
Huo	2013	China	Asian	187/221	408	HB	OC	MassARRAY	7
Vangsted	2012	Denmark	Caucasian	328/1696	2024	PB	MM	Taqman	6
Zhou	2009	China	Asian	163/203	366	HB	NPC	PCR-PAGE	8
Han	2015	China	Asian	936/936	1872	PB	PC	PCR-RFLP	8
Li	2015	China	Asian	220/222	442	HB	Osteosarcoma	PCR-RFLP	7
Liu	2015	China	Asian	906/1072	1978	HB	NPC	TaqMan	7
Liu	2015	China	Asian	684/907	1591	HB	NPC	TaqMan	7
Wang	2015	China	Asian	421/425	846	HB	LC	PCR-RFLP	8
Lu	2015	China	Asian	687/687	1374	HB	OC	PCR-RFLP	7
Kopp	2015	Denmark	Caucasian	1,010/1,829	2634	PB	CRC	PCR-KASP	6
Chen	2015	China	Asian	411/438	852	HB	OC	MassARRAY	7
Zhang	2014	China	Asian	208/1606	2230	HB	HCC	PCR-RFLP	6
Hua	2014	China	Asian	401/433	834	HB	GC	MassARRAY	8
Wang	2015	China	Asian	352/459	811	HB	PTC	PCR-PAGE	8
Li	2015	China	Asian	730/780	1510	HB	BC	TaqMan	8
Li	2015	China	Asian	1216/1588	2804	HB	RCC	TaqMan	8
Li	2015	China	Asian	820/945	1765	HB	PC	TaqMan	8
Rybka	2016	Poland	Caucasian	62/126	188	NA	AML	PCR-CGE	6
Eskandari	2016	Iran	Asian	236/203	439	PB	BC	AS-PCR	9
Zhang	2015	China	Asian	718/718	1436	PB	LC	PCR-RFLP	8

*AS-PCR,* Allele-Specific polymerase chain reaction*; PCR-CGE,* polymerase chain reaction with capillary gel electrophoresis; *PCR-RFLP*, polymerase chain reaction-restriction fragment length polymorphism; *PCR-PAGE*, polymerase chain reaction-polyacrylamide gel electrophoresis; *HB*, hospital-based study; *PB*, population-based study; *NA*, not available; *BC*, bladder cancer; *PC*, prostate cancer; *OC*, ovarian cancer; *LC*, lung cancer; *HCC*, hepatocellular carcinoma; *MM*, multiple myeloma; *OSCC*, oral squamous cell carcinoma; *CSCC*, cervical squamous cell carcinoma; *CRC*, colorectal cancer; *NPC*, nasopharyngeal carcinoma; *GC*, gastric cancer; *GNT*, gastroenteropancreatic neuroendocrine tumor; *HNSCC*, squamous cell carcinomas of the head and neck region; *BCLL*, B cell chronic lymphocytic leukemia; *RCC*, renal cell carcinoma; *ESCC*, esophageal squamous cell carcinoma; *BRC*, breast cancer; *PTC*, papillary thyroid carcinoma; *AML*, acute myeloid leukaemia.

**Table 2:** Distributions of NF-κB1-94 ins/del ATTG promoter polymorphism allele and genotypes in different groups.

Author	Case					Control					
	DD	DW	WW	D (%)	W (%)	DD	DW	WW	D (%)	W (%)	HWE
Lin	50	103	59	203 (47.9)	221 (52.1)	58	100	43	216 (53.7)	186 (46.3)	0.993
Riemann	27	58	54	112 (40.3)	166 (59.7)	48	141	118	237 (38.6)	377 (61.4)	0.586
Riemann	13	41	18	67 (46.5)	77 (53.5)	48	141	118	237 (38.6)	377 (61.4)	0.586
Riemann	17	76	47	110 (39.3)	170 (60.7)	48	141	118	237 (38.6)	377 (61.4)	0.586
Riemann	30	124	88	184 (38)	300 (62)	48	141	118	237 (38.6)	377 (61.4)	0.586
Lehnerdt	53	179	132	285 (39.2)	443 (60.8)	48	141	118	237 (38.6)	377 (61.4)	0.58
Lo	31	89	62	151 (41.5)	213 (58.5)	34	62	20	130 (56)	102 (44)	0.361
He	35	84	83	154 (38.1)	250 (61.9)	124	183	97	431 (53.3)	377 (46.7)	0.07
Zhang	14	57	46	85 (36.3)	149 (63.7)	31	68	44	130 (45.5)	156 (54.5)	0.624
Zhou	20	105	108	145 (31.1)	321 (68.9)	64	166	135	294 (40.3)	436 (59.7)	0.297
Andersen	62	195	121	319 (42.2)	437 (57.8)	102	347	307	551 (36.4)	961 (63.6)	0.801
Tang	26	92	89	144 (34.8)	270 (65.2)	46	108	74	200 (43.9)	256 (56.1)	0.565
Song	138	500	363	776 (38.8)	1226 (61.2)	186	522	297	894 (44.5)	1116 (55.5)	0.102
Fan	17	84	78	118 (33)	240 (67)	44	103	76	191 (42.8)	255 (57.2)	0.396
Lin	100	246	116	446 (48.3)	478 (51.7)	168	271	81	607 (58.4)	433 (41.6)	0.099
Ungerback	43	187	114	273 (39.7)	415 (60.3)	96	270	256	462 (37.1)	782 (62.9)	0.079
Huang	225	459	372	909 (43)	1203 (57)	210	491	355	911 (43.1)	1201 (56.9)	0.089
Huang	104	230	169	438 (43.5)	568 (56.5)	145	289	189	579 (46.5)	667 (53.5)	0.092
Cheng	29	64	42	122 (45.2)	148 (54.8)	168	271	81	607 (58.4)	433 (41.6)	0.099
Li	151	269	189	571 (46.9)	647 (53.1)	93	324	223	510 (39.8)	770 (60.2)	0.156
Oltulu	16	44	35	76 (40)	114 (60)	6	47	46	59 (29.8)	139 (70.2)	0.18
Kopp	54	152	128	260 (38.9)	408 (61.1)	64	161	109	289 (43.3)	379 (56.7)	0.741
Huo	22	82	83	126 (33.7)	248 (66.3)	47	103	71	197 (44.6)	245 (55.4)	0.399
Vangsted	55	163	110	273 (41.6)	383 (58.4)	253	778	665	1284 (37.9)	2108 (62.1)	0.303
Zhou	22	67	74	111 (34.1)	215 (62.9)	42	90	71	174 (42.9)	232 (57.1)	0.177
Han	534	339	63	1407 (75)	465 (25)	567	331	38	1465 (78.3)	407 (21.7)	0.23
Li	46	114	60	206 (46.8)	234 (53.2)	66	106	50	238 (53.6)	206 (46.4)	0.55
Liu	152	438	316	742 (41)	1070 (59)	224	512	336	960 (44.8)	1184 (55.2)	0.262
Liu	117	331	236	565 (41)	803 (59)	195	438	274	828 (45.6)	986 (54.4)	0.169
Wang	89	219	113	397 (47.2)	445 (52.8)	131	205	89	467 (54.9)	383 (45.1)	0.595
Lu	221	351	115	793 (57.7)	581 (42.3)	253	339	95	845 (61.5)	529 (38.5)	0.271
Kopp	146	449	320	741 (40.5)	1089 (59.5)	253	787	679	1293 (37.6)	2145 (62.4)	0.311
Chen	95	195	120	385 (47)	435 (53)	122	235	85	479 (54.2)	405 (45.8)	0.136
Zhang	107	312	205	526 (42.2)	722 (57.8)	274	790	542	1338 (41.7)	1874 (58.3)	0.631
Hua	127	182	92	436 (54.4)	366 (45.6)	83	230	120	396 (45.7)	470 (54.3)	0.144
Wang	60	186	106	306 (43.5)	398 (56.5)	79	209	171	367 (40)	551 (60)	0.273
Li	187	316	227	690 (47.3)	770 (52.7)	124	395	261	643 (41.2)	917 (58.8)	0.208
Li	188	577	451	953 (39.2)	1479 (60.8)	225	781	582	1231 (38.8)	1945 (61.2)	0.152
Li	144	377	299	665 (40.6)	975 (59.4)	136	462	347	734 (38.8)	1156 (61.2)	0.371
Rybka	7	30	25	44 (35.5)	80 (64.5)	14	69	43	97 (38.5)	155 (61.5)	0.08
Eskandari	18	122	96	158 (33.5)	314 (66.5)	35	106	62	176 (43.3)	230 (56.7)	0.37
Zhang	32	252	434	316 (22)	1120 (78)	76	290	352	442 (30.8)	994 (69.2)	0.16

*HWE*, Hardy-Weinberg equilibrium.

*WW*, homozygous insertion or wild-type; *DD*, homozygous deletion or variant; *DW*, heterozygous ins/del.

**Table 3:** Summary of overall results and subgroup for the association between the NF-κB1-94 ins/del ATTG promoter polymorphism and cancer risk.

	No.	Sample size (N)		DD+DI vs. II		DD vs. DI+II		DD vs. II		DI vs. II		D vs. I	
		Case	Control	OR (95% CI)	P_OR_ a	OR (95% CI)	P_OR_ a	OR (95% CI)	P_OR_ a	OR (95% CI)	P_OR_ a	OR (95% CI)	P_OR_ a
Overall	42	18,222	24,778	**0.86 (0.79–0.95)**	**0.002**	**0.84 (0.74–0.94)**	**0.003**	**0.77 (0.66–0.90)**	**0.001**	**0.90 (0.83–0.98)**	**0.011**	**0.89 (0.83–0.96)**	**0.002**
Control source													
PB	10	5,369	8,212	0.90 (0.72–1.13)	0.354	0.77 (0.62–0.96)	0.02	0.72 (0.52–1.01)	0.053	0.96 (0.78–1.17)	0.689	0.89 (0.76–1.04)	0.15
HB	29	12,463	15,976	**0.85 (0.77–0.95)**	**0.003**	**0.86 (0.74–0.99)**	**0.04**	**0.78 (0.65–0.93)**	**0.007**	**0.88 (0.81–0.96)**	**0.004**	**0.89 (0.81–0.97)**	****0.006****
Ethnicity													
Caucasian	12	3,413	6,887	**1.20 (1.1–1.31)**	**<0.0001**	1.03 (0.92–1.16)	0.583	**1.15 (1.01–1.31)**	**0.03**	**1.22 (1.11–1.34)**	**<0.0001**	**1.11 (1.04–1.17)**	**0.001**
Asian	30	14,809	17,891	**0.77 (0.70–0.86)**	**<0.0001**	**0.77 (0.67–0.90)**	**0.001**	**0.66 (0.55–0.80)**	**<0.0001**	**0.85 (0.81–0.90)**	**<0.0001**	**0.82 (0.76–0.90)**	**<0.0001**
Genotyping methods													
PCR-RFLP	11	5,450	6,525	**0.75 (0.62–0.90)**	**0.002**	**0.72 (0.60–0.87)**	**0.001**	**0.62 (0.47–0.82)**	**0.001**	**0.81 (0.70–0.94)**	**0.005**	**0.87 (0.80–0.95)**	**0.001**
MassARRAY	3	998	1,096	0.77 (0.44–1.32)	0.334	0.93 (0.43–2.01)	0.862	0.77 (0.29–2.07)	0.608	0.75 (0.53–1.07)	0.111	0.87 (0.80–0.95)	0.609
TaqMan	14	8,505	12,059	0.95 (0.83–1.09)	0.446	0.99 (0.83–1.21)	0.977	0.95 (0.76–1.19)	0.654	0.95 (0.84–1.07)	0.375	0.87 (0.80–0.95)	0.685
PCR-PAGE	6	1,284	1,599		**0.08**	**0.65 (0.50–0.85)**	**0.002**	**0.58 (0.39–0.86)**	**0.008**	0.86 (0.66–1.12)	0.257	**0.87 (0.80–0.95)**	**0.015**
PCR-CGE	2	241	349	0.70 (0.50–0.98)	0.037	0.61 (0.26–1.40)	0.239	0.51 (0.23–1.13)	0.096	0.78 (0.55–1.12)	0.174	0.87 (0.80–0.95)	0.017
Pyrosequencing	4	593	1,228	1.18 (0.94–1.48)	0.146	0.96 (0.72–1.28)	0.756	1.06 (0.78–1.45)	0.693	1.23 (0.94–1.60)	0.125	0.87 (0.80–0.95)	0.364
Quality score													
<7 points	24	8,849	13,548		**0.013**	**0.81 (0.71–0.91)**	**0.001**	**0.75 (0.63–0.89)**	**0.001**	0.91 (0.82–1.01)	0.076	**0.87 (0.80–0.95)**	**0.002**
>7 points	18	9,373	11,230	0.87 (0.74–1.01)	0.066	0.87 (0.70–1.08)	0.205	0.79 (0.60–1.04)	0.086	0.89 (0.78–1.01)	0.073	0.91 (0.80–1.03)	0.126
Cancer Type													
OSCC	2	674	721	**0.60 (0.46–0.77)**	**<0.0001**	**0.63 (0.49–0.81)**	**<0.0001**	**0.49 (0.33–0.72)**	**<0.0001**	**0.67 (0.51–0.88)**	**0.004**	**0.70 (0.60–0.82)**	**<0.0001**
CRC	5	2,777	4,409	1.12 (0.85–1.48)	0.409	0.98 (0.76–1.26)	0.849	1.06 (0.74–1.52)	0.768	1.14 (0.87–1.49)	0.336	1.06 (0.88–1.27)	0.558
BC	4	1,788	1,955		0.884	1.16 (0.68–1.97)	0.578	1.12 (0.64–1.96)	0.692	0.95 (0.81–1.11)	0.48	1.05 (0.82–1.36)	0.686
GC	2	583	549	0.74 (0.24–2.31)	0.603	1.00 (0.26–3.85)	0.996	0.78 (0.12–5.12)	0.799	0.72 (0.33–1.57)	0.41	0.90 (0.34–2.24)	0.814
HCC	3	961	2,530	0.59 (0.30–1.15)	0.119	0.67 (0.40–1.10)	0.114	0.50 (0.21–1.16)	0.106	0.65 (0.37–1.15)	0.137	0.69 (0.44–1.09)	0.114
PC	4	2,207	2,358	0.79 (0.61–1.02)	0.074	0.89 (0.66–1.20)	0.447	0.72 (0.45–1.16)	0.177	0.84 (0.71–1.00)	0.05	0.88 (0.74–1.05)	0.149
OC	4	1,463	1,573	**0.67 (0.56–0.79)**	**<0.0001**	**0.68 (0.52–0.89)**	**0.005**	**0.54 (0.40–0.73)**	**<0.0001**	**0.73 (0.61–0.87)**	**0.001**	**0.75 (0.65–0.86)**	**<0.0001**
LC	5	2,793	2,921	0.83 (0.67–1.03)	0.092	0.82 (0.54–1.26)	0.368	0.77 (0.48–1.26)	0.302	**0.84 (0.74–0.96)**	**0.008**	0.88 (0.70–1.09)	0.246
NPC	3	1,573	2,182	**0.82 (0.72–0.93)**	**0.003**	**0.74 (0.63–0.88)**	**<0.0001**	**0.63 (0.49–0.81)**	**<0.0001**	0.63 (0.49–0.81)	0.067	**0.83 (0.76–0.91)**	**<0.0001**
RCC	2	1,356	1,895	1.01 (0.87–1.17)	0.892	1.01 (0.72–1.40)	0.973	1.06 (0.85–1.31)	0.625	1.07 (0.78–1.47)	0.688	1.02 (0.92–1.13)	0.71
Other cancers	8	1,867	3,685	0.99 (0.78–1.27)	0.962	0.79 (0.59–1.05)	0.1	0.81 (0.55–1.21)	0.307	1.07 (0.86–1.31)	0.553	0.93 (0.77–1.13)	0.468

*OR*, odds ratio; *CI*, confidence interval.

*P* values in bold denotes significance.

^a^The *P* values of Z test for odds ratios test.

**Table 4:** Summary of the corrected *P* value for multiple testing and heterogeneity test in this meta-analysis.

	No.	Sample size (N)		DD+DI vs. II			DD vs. DI+II			DD vs. II			DI vs. II			D vs. I		
		Case	Control	P_Corr_^a^	I^2^(%)^b^	P_Het_ c	P_Corr_^a^	I^2^(%)^b^	P_Het_ c	P_Corr_^a^	I^2^(%)^b^	P_Het_ c	P_Corr_^a^	I^2^(%)^b^	P_Het_ c	P_Corr_^a^	I^2^(%)^b^	P_Het_^c^
Overall	42	18,222	24,778	**0.005**	77.1	<0.0001	**0.0037**	80.2	<0.0001	**0.005**	83.8	<0.0001	**0.011**	64.1	<0.0001	**0.005**	84	<0.0001
Control source																		
PB	10	5,369	8,212	0.443	86.6	<0.0001	0.1	81.5	<0.0001	0.133	83.1	<0.0001	0.689	71.3	<0.0001	0.25	87.8	<0.0001
HB	29	12,463	15,976	**0.015**	73.3	<0.0001	**0.04**	77.5	<0.0001	**0.0088**	79.1	<0.0001	**0.01**	53.9	<0.0001	**0.01**	81.2	<0.0001
Ethnicity																		
Caucasian	12	3,413	6,887	**<0.001**	35.3	0.108	0.583	21.8	0.229	**0.0375**	31.9	0.136	**<0.001**	32.8	0.128	**0.0017**	32	0.135
Asian	30	14,809	17,891	**<0.001**	74	<0.0001	**0.001**	84.5	<0.0001	**<0.001**	86.3	<0.0001	**<0.001**	46.5	0.003	**<0.001**	81.1	<0.0001
Genotyping methods																		
PCR-RFLP	11	5,450	6,525	**0.0025**	73.6	<0.0001	**0.003**	71.9	<0.0001	**0.005**	78.9	<0.0001	**0.005**	55.7	0.012	**0.002**	80.6	<0.0001
MassARRAY	3	998	1,096	0.835	86.7	0.001	0.862	89	<0.0001	1.000	89.1	<0.0001	0.555	65	0.057	0.761	88	<0.0001
TaqMan	14	8,505	12,059	1.000	78.4	<0.0001	0.977	84.2	<0.0001	1.000	85.1	<0.0001	1.000	69	<0.0001	0.856	85.2	<0.0001
PCR-PAGE	6	1,284	1,599	**0.1**	70.1	0.005	**0.01**	39.5	0.142	**0.02**	67.1	0.01	0.257	58.6	0.034	**0.025**	72.1	0.003
PCR-CGE	2	241	349	0.0925	0	0.722	0.239	55.7	0.133	0.16	43.6	0.183	0.218	0	0.879	0.085	13.4	0.283
Pyrosequencing	4	593	1,228	0.365	12.4	0.331	0.756	6.9	0.358	0.866	0	0.402	0.625	27.7	0.246	0.607	0	0.472
Quality score																		
<7 points	24	8,849	13,548	**0.016**	71.6	<0.0001	**0.005**	61	<0.0001	**0.003**	74.5	<0.0001	0.076	57.5	<0.0001	**0.0033**	76.7	<0.0001
>7 points	18	9,373	11,230	0.33	78.9	<0.0001	0.205	82	<0.0001	0.143	85.3	<0.0001	0.183	67.5	<0.0001	0.158	87	<0.0001
Cancer Type																		
OSCC	2	674	721	**<0.001**	0	0.377	**<0.001**	3.9	0.308	**<0.001**	33	0.222	**0.004**	0	0.57	**<0.001**	6.9	0.3
CRC	5	2,777	4,409	1.000	85	<0.0001	0.849	68.3	0.013	0.96	81.4	<0.0001	1.000	82.1	<0.0001	0.93	84.4	<0.0001
BC	4	1,788	1,955	0.884	60.8	0.054	1.000	88.4	<0.0001	0.865	87.1	<0.0001	1.000	11.3	0.336	0.966	85.7	<0.0001
GC	2	583	549	1.000	84.4	<0.0001	0.996	90.3	<0.0001	1.000	91.4	<0.0001	1.000	80.9	0.022	1.000	91.6	<0.0001
HCC	3	961	2,530	0.149	92.2	<0.0001	0.285	82.4	0.003	0.53	89.9	<0.0001	0.137	85.5	<0.0001	0.19	89.8	<0.0001
PC	4	2,207	2,358	0.185	57.6	0.07	0.447	70	0.019	0.221	78.2	0.003	0.25	9.6	0.345	0.248	69.5	0.02
OC	4	1,463	1,573	**<0.001**	0	0.462	**0.005**	51.6	0.102	**<0.001**	39.8	0.173	**0.0012**	0	0.416	**<0.001**	38.5	0.181
LC	5	2,793	2,921	0.23	69.1	0.012	0.368	86.3	<0.0001	0.378	86.9	<0.0001	**0.04**	12	0.337	0.41	83.2	<0.0001
NPC	3	1,573	2,182	**0.0038**	0	0.504	**<0.001**	0	0.728	**<0.001**	0	0.561	0.067	0	0.633	**<0.001**	0	0.426
RCC	2	1,356	1,895	1.000	1.9	0.313	0.973	34.2	0.218	1.000	0	0.583	1.000	53.6	0.142	1.000	0	0.945
Other cancers	8	1,867	3,685	0.962	71.8	0.001	0.500	53.2	0.046	0.768	76.4	0.002	0.691	56.4	0.025	0.78	77.6	<0.0001

*P* values in bold denotes significance.

^a^*P* Values were corrected to adjust for multiple testing.

^b^The value of *I*^*2*^ statistics for heterogeneity test.

^c^*P* value of the Q-test for heterogeneity test.
